# A practical framework to approach the development and evaluation of patient registries for rare diseases

**DOI:** 10.1186/s13023-026-04270-0

**Published:** 2026-02-24

**Authors:** Maya S Vaishnaw, Rachel Richesson

**Affiliations:** 1 Department of Medical Genetics and Genomics, Cleveland Clinic Foundation, 9500 Euclid Avenue, Cleveland, OH 44195 USA; 2 Department of Learning Health Sciences, University of Michigan Medical School, Victor Vaughan Building 2054, 1111 Catherine Street, Ann Arbor, MI 48109-2054 USA

## Abstract

**Background:**

Patient registries are essential to rare disease research, but the implementation and maintenance of a registry requires substantial investments. Despite the large number of rare disease communities that currently have, or are planning for, a registry, there is little guidance on if and when a rare disease registry should be established. There is also little guidance on how to articulate registry objectives to address current and evolving scientific and community needs.

**Main Body:**

A practical framework is needed to assist rare disease registry stakeholders to understand and prioritize registry objectives and requirements for registry design, data collection, and funding. In this review, we synthesize existing patient registry classifications and prior reports of rare disease registry experience into a unified framework characterizing rare disease registries by their types of research questions and scientific needs.

**Conclusion:**

This simple framework can be used to help rare disease communities identify their research requirements, and support decision making around whether to initiate, and how invest in, registry infrastructure.

## Background

Rare diseases are globally defined in terms of their impact on small populations. In the United States, a disorder is considered rare if it affects fewer than 200,000 people, while in the European Union, rare disease is defined as affecting 1in 2000 individuals. [1, 2] Despite variation in definition, there are estimated to be over 10,000 rare diseases that collectively represent a substantial impact on patients and public health. The use of patient registries to collect historical and prospective data is a common approach to understand specific rare conditions. Rare disease registries have proven critical over the last 30 years, aggregating data to support clinical and translational research for affected patients and a diverse range of stakeholders [3–10].

Although there is no single authoritative classification or inventory of registries, the number of unique rare disease registries is likely in the thousands. Rare disease registries have been developed by motivated patients and their families, advocacy groups, clinicians, national health systems, and biopharmaceutical product manufacturers. [10] The value of rare disease registries to a range of stakeholders evidenced by the diversity of rare disease registry sponsors spanning nonprofit organizations and biopharmaceutical companies. Rare disease registries have been used to address a wide range of purposes, including: estimation of disease prevalence or incidence, understanding disease etiology, planning for population health and health care delivery services, diagnostic classification, operation and evaluation of services, evaluation of treatment patterns, decisions around insurance coverage and reimbursement, and clinical quality improvement (e.g., Cystic Fibrosis Foundation Patient Registry [11]).

More commonly, rare disease registries are developed to directly support clinical research needs, including patient recruitment, observational research, clinical trial design, and more recently, as direct sources of real world data (RWD) for observational studies and clinical trials, including for historical control arms. [12] Given the importance of registries to rare disease research, the cumulative prevalence of rare diseases, and technological and methodological innovations around the integration of real world clinical data from electronic health record systems, the need for and interest in building registries will continue to increase.[4, 13]

Anecdotally, the authors have witnessed many patient advocacy groups aspiring and raising funds for developing registries. However, the costs for developing registry programs are considerable. The complexities of observational research methods, data management and analysis, international facilitation, and diverse stakeholder interests require continual management and governance and funding. Hence, careful consideration should be given before developing a registry program initiative.[10, 13]

Even before the digital health transformations over past several decades, a foundational review of registries in 1973 recommended that the value of all registries be examined regularly to ensure that their objectives are relevant and being addressed. [14] The review further suggested that sponsors interrogate whether their research objectives could be met in any other way; and if the answer is yes, then a registry is likely to be an inefficient use of resources. Despite this longstanding recommendation, there is little guidance for how and when to design, build, and maintain rare disease registries. [15, 16] Although some guidelines have been developed, such as the European Medicines Agency (EMA) guidance on use of registry data for research investigations [17], there are no guidelines for how to determine if a rare disease registry should be built at all or for how to articulate registry goals that are both realistic and relevant to scientific and community needs over time. A meaningful framework to assess and prioritize data collection, funding, and registry design needs in this complex landscape is long overdue and urgently needed.

## Objectives

The objective of this review is to synthesize information from guidance documents and experience reports into a simple framework that can inform registry stakeholder decision-making and registry planning to advance research goals.

## Methods

We reviewed relevant informatics book chapters and guidance documents, review articles on registries, and case reports from mature registry programs to identify general data requirements of rare disease registries used for different purposes. We mapped the different registry purposes to each of the five iterative stages of translational science, basic research (T0), preclinical research (T1, translation to humans), clinical research (T2, translation to patients), clinical implementation (T3, translation to practice), and public health (T4,translation to community). [18] We then used commonly observed research program milestones to organize registry purposes into groups by broad research objective, and identified research goals, research questions, and data requirements for each group of related registry purposes.

### Characterizing rare disease registry goals with scientific advancements

Previous characterizations of rare disease registries recognize a range of biomedical research objectives, including to understand the etiology of rare diseases, to learn the natural history, evolution, risk, and outcomes of specific diseases, and to develop a patient base for evaluating drugs, medical devices, and orphan products.[10]

**Fig. 1:**
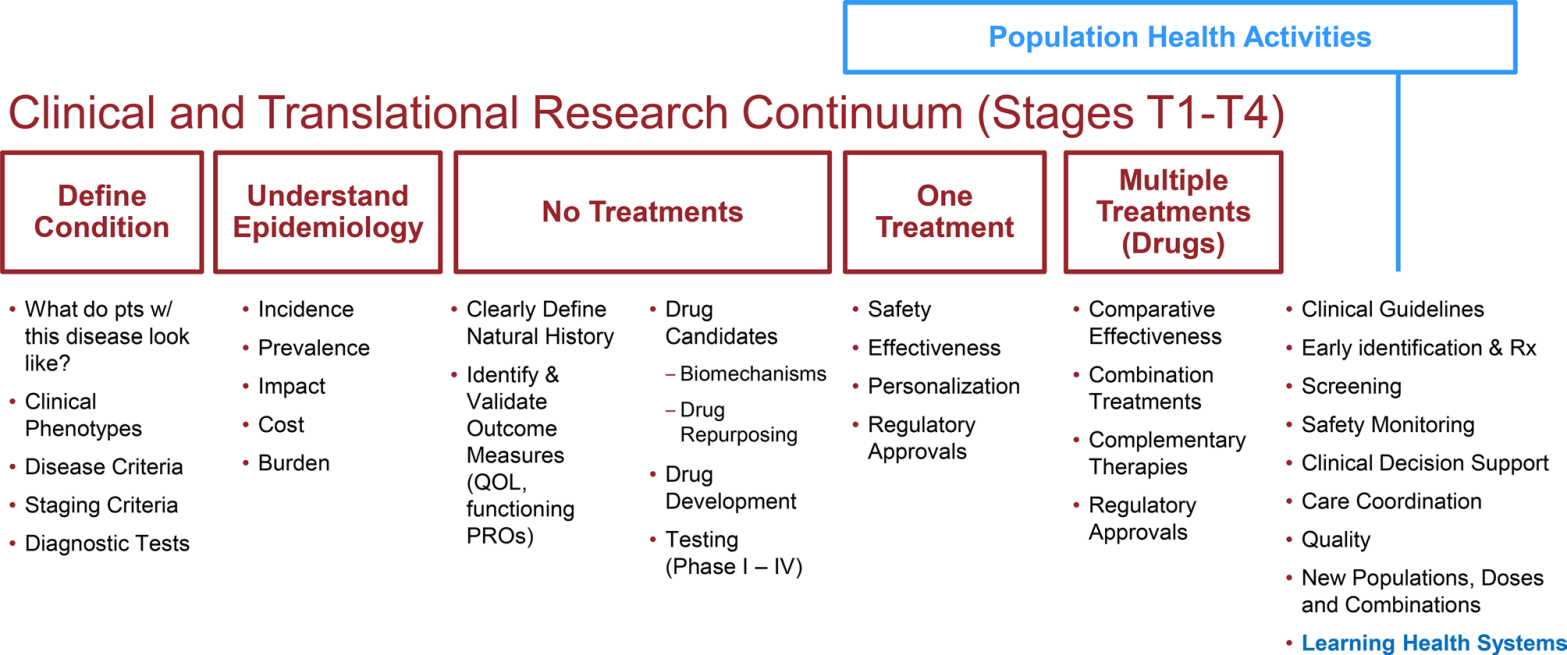
Possible registry goals and objectives, organized by the state of the scientific and medical knowledge of the condition on the translational research continuum (shown in red)

We provide a simple visual representation (Fig. 1) that relates the state of the scientific and medical knowledge about a rare disease to outstanding research needs and research questions. This figure illustrates for example, that if the state of science reflects gaps in understanding the epidemiology of a rare condition of interest, then a rare disease registry might be needed to support research questions around incidence, prevalence, and impact. In contrast, if one or more therapeutics exist for a condition, the registry could be used to support comparative effectiveness studies, combination therapies, or population health initiatives. Figure 1 thereby also illustrates how knowledge gaps in understanding disease and development of therapeutics, and associated research questions, can be used to define immediate rare disease registry purpose and objectives.

Although research and discovery does not always progress linearly, we chose to present the states of scientific advancement sequentially, as this is a common model for biomedical research programs (i.e., the disorder is first identified, diagnostic criteria are defined, the underlying cause is characterized, natural history and public health burden are assessed, and treatments are then developed, approved and optimized). Registries can be used to support all of these activities in this general pattern of scientific discovery and clinical care.[11]

### Examples from LSDs and development of enzyme replacement therapies

To illustrate the role of registries in this general pattern of scientific discovery and growth of medical knowledge, we use the research experience of rare lysosomal storage disorders as a prototypic example. Gaucher disease is a rare, genetic, lysosomal storage disorder caused by deficiency of the enzyme glucocerebrosidase. The International Collaborative Gaucher Group’s (ICGG) rare disease registry was established over 30 years ago coincident with the development of enzyme replacement therapy, supported by Genzyme corporation. [5] The ICGG was designed to characterize long term treatment efficacy, but has enabled study of disease natural history and therapeutic endpoints over time.[4]

Using the ICGG collaboration and registry as a model, registries for other lysosomal storage disorders (e.g., Fabry, Mucopolysaccharidosis type I, and Pompe diseases) were subsequently created alongside the development of corresponding enzyme replacement therapies and increased biopharmaceutical funding. Mistry et al. present an excellent summary of the history and evolving needs of these lysosomal storage disorder registries, including to fulfill regulatory commitments related to therapeutic development and to provide a better understanding of rare disorder natural history and long-term treatment efficacy. Collectively, these lysosomal storage disorder registries illustrate how rare disease registry data collection needs cannot be solely categorized by a solitary and fixed primary objective, and also how registries can support multiple purposes with progress through the translational research stages.

### A chronological framework for identifying scientific needs and registry requirements

**Fig. 2:**
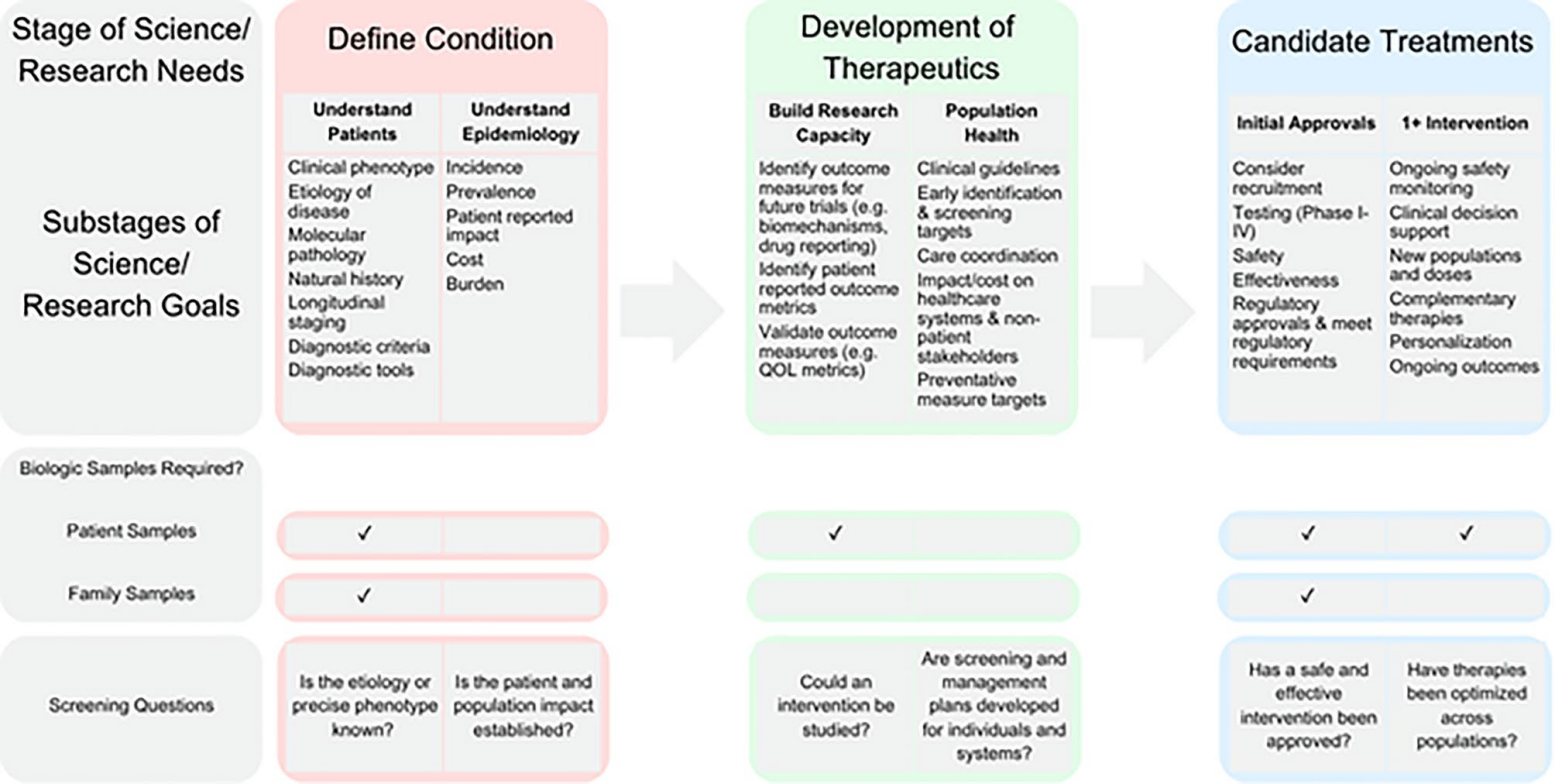
Rare disease registry requirements for biological samples guided by stage of research and research goals

We expand on the valuable work of Mistry et al. and others to connect scientific needs to registry design and data requirements. While every rare disease registry should have a clearly defined purpose, lysosomal storage disease registries modeled on the initial IGGG show that functional distinctions in data collection are not based on one static, primary, goal, but instead on multifaceted needs of different stages in the rare disease research pipeline. We provide a more operational framework (Fig. 2) to understand how registry initiatives can support different chronological stages of research and associated research goals.

Rare disease registry design requirements can be organized broadly by stage and sub-stage of science and outstanding research needs, based on primary need to: 1) define the condition, 2) develop therapeutics, and 3) provide and evaluate candidate treatments (Fig. 2). Each of these stages can be further divided into substages defined by relevant research goals or objectives (e.g., to understand patients, epidemiology, build research capacity, improve population health). These research objectives can encompass specific disease-relevant research questions and subsequent requirements for biological samples and data. Possible screening questions (e.g., is the etiology or precise phenotype known? Can an intervention be studied? Has a safe & effective intervention been approved?) to help users assess the state of the science and possible registry and research goals for their condition. Together, the scientific priorities, research goals and registry requirements can help registry sponsors to prioritize requirements and resources for registry governance, design, implementation, and growth.

### Characterization of registry purpose to guide data requirements

Different types of registries have distinct data needs. Table 1, used with permission from Springer, presents a characterization of patient registries by broad purpose and suggests essential data requirements for each. [19]

**Table 1:** Essential data requirements by broad purpose of patient registry [19]

Essential requirements				
**Purpose**	**Completeness of case ascertainment**	**Clinical data**^**a**^ **(beyond diagnosis or procedure)**	**Verification of data validity**	**Follow-up data**
*Public health (“population-based”)*				
Population surveillance	Yes	No	Yes	No
Contact notification	Yes	No	No	No
Patient compliance (for management of infectious diseases)	Yes	Yes	Yes	Yes
Planning (community and service)	Yes	No	No	No
Policy	Yes	No	No	No
*Health services research*				
Evaluation of healthcare/education delivery	Yes	Yes	Yes	No
Facilitate health utilization treatment patterns	Yes	No	Yes	Yes
Monitoring health services	Yes	No	No	No
Measuring healthcare quality	No	Yes	Yes	Yes
*Health promotion tools and education*				
Patient education notifications	No	No	No	No
Physician education notifications	No	No	No	No
Aggregate data for patient education/support	No	No	No	No
*Patient care*				
Chronic disease management	No	Yes	Yes	Yes
Vaccination	Yes	No	No	Yes
*Clinical research - funding and support*				
Research funding decisions	No	No	No	No
Research planning and design	No	No	No	No
Cohort selection	No	Yes	Yes	No
Recruitment - outreach to patients	No	No	No	No
*Clinical research - scientific inquiry*				
Cross-sectional	Yes	Yes	Yes	No
Longitudinal	Yes	Yes	Yes	Yes
*Regulatory*				
Safety of agents (postmarketing)	Yes	Yes	Yes	Yes
Efficacy of agents (postmarketing; phase 4)	Yes	Yes	Yes	Yes

Clinical data - additional data beyond the data elements required for determining eligibility for the registry. Eligibility is determined either by disease, exposure, or patient characteristics

This table illustrates the variation in data collection and design requirements relative to specific registry objectives and essential functionality. A clear articulation of registry purpose (from Figs. 1 & 2) can help registry sponsors and developers to identify specific data requirements for different (scientific and biomedical) research needs and registry goals [19]. For example, complete case ascertainment might be critical for some registry purposes (e.g., population surveillance) and not essential for others (e.g., chronic disease management, research planning).

### Implementation implications

We suggest stakeholders use our conceptualization of rare disease research objectives by translational science continuum (Fig. 1) to identify the appropriate research stage and efficiently develop registry goals and associated design requirements. Our framework can be used to highlight what types of data are needed for different research purposes and stages, thereby enabling rare disease registry designers to prioritize data collection and resource allocation to address the outstanding research questions at each stage (Fig. 2). Every rare disease is different in terms of what is known and what resources are available, and what types of data collection are essential and/or feasible to collect. We see this framework as a guide – not a prescriptive algorithm for registry design or decisions. The framework can be applied to various types of rare diseases, including those without existing treatment pipelines or with strong patient-led infrastructure.

Because this framework includes a range of rare disease research milestones and the stages of translational science, it facilitates a forward-looking approach to designing and growing registry programs. Successful understanding of the nature and epidemiology of a disease is foundational to the discovery and development of treatment interventions. A registry developed to address one set of research goals can be designed to accommodate future translational stages and research needs. For example, a registry might first support an inventory of cases to estimate prevalence and features of the conditions for a rare disease that is not yet characterized, may later be used to test drug targets and candidates, and eventually support population level safety monitoring in the future.

The overlapping and evolving state of the science, research objectives, and motivations for rare disease registries provide a framework that can support decision making for design and data collection in rare disease registries over time. Further, the multi-purpose and shifting requirements of rare disease registries, in the context of scientific advances and international regulatory oversight, make it challenging for sponsors, advocates, and scientists to navigate the complex, dynamic, and open ecosystem of research, technology, and health care delivery. Potential registry sponsors will benefit from a versatile framework to approach funding and design of registries based on the needs of their specific rare disease community across the translational science continuum.

Given the limitations in both resources and sample sizes that are inherent for all rare diseases, the re-use of existing data and infrastructure should be considered at all stages. As rare disease research programs advance to address population health activities (e.g., drug safety monitoring, clinical guideline development, quality assessment), standardized clinical data and patient contributed data will be needed. [5, 20] As quality of life measures and patient reported outcomes become increasingly important in rare disease best practice guidelines and regulatory approvals, patient reported data collection should be considered in initial rare disease registry design. [5, 21] Leveraging relevant guidelines from the EMA and the United States Food and Drug Administration (FDA) Real-World Evidence Programs [22, 23] to shape study design from early stages will encourage best practices and facilitates use of rare disease registry data in downstream regulatory processes.

## Conclusions

Using the translational research stages and state of the science to identify and articulate rare disease registry objectives and data needs can inform data needs assessment, financial investment, and decision making for current and planned rare disease registry initiatives. A simplified chronological view of the evolution of scientific knowledge and research objectives can provide a structured framework to define key data collection priorities across research stages. By applying this framework, future projects and funding allocation may be systematically developed and assessed. Additionally, this characterization has the potential to broadly define priorities when establishing a health information technology structure, enabling decision making around whether and how to and develop a registry to support research for a given rare condition. We propose the use of this framework to address these critically important development and evaluation needs for sponsors and stakeholders of rare disease registries, across the spectrum of research stages, conditions, and stakeholder requirements.

## Data Availability

Not applicable.
